# Uncommon serum creatine phosphokinase and lactic dehydrogenase increase during diosmin therapy: two case reports

**DOI:** 10.1186/1752-1947-8-194

**Published:** 2014-06-16

**Authors:** Giulia Milano, Silvia Leone, Carmen Fucile, Maria Laura Zuccoli, Andrea Stimamiglio, Antonietta Martelli, Francesca Mattioli

**Affiliations:** 1 Department of Internal Medicine, Clinical Pharmacology and Toxicology Unit, University of Genoa, Viale Benedetto XV 2, Genoa I-16132, Italy; 2 General Practitioner – Via Struppa, 256 r, Genoa I-16138, Italy

**Keywords:** Chronic venous insufficiency, Creatine phosphokinase, Diosmin, Lactic dehydrogenase

## Abstract

**Introduction:**

Short-term administration of diosmin is usually considered safe, with only minor side effects (stomach and abdominal pain, diarrhea, dermatological disorders, and headache) occasionally observed. Within a 4-year period, a general practitioner noticed 17 cases of mild, diosmin-induced side effects, two of which showed particular interest.

**Cases presentation:**

Case 1: A 55-year-old Caucasian woman presented with chronic leg venous insufficiency. She was prescribed diosmin 450mg twice a day. After 5 days of therapy, she developed pain in the legs (myalgia), and diosmin therapy was suspended. She made a spontaneous attempt of drug rechallenge and her leg pain reappeared. Thus, she underwent blood analysis, which showed elevation of creatine phosphokinase levels. Creatine phosphokinase values normalized only after prolonged discontinuation of the therapy. Case 2: A 79-year-old Caucasian man, who was diagnosed with acute hemorrhoidal syndrome. After 21 days of continuous diosmin treatment, increased levels of serum lactic dehydrogenase were detected. In both cases a comprehensive analysis of all possible causes for enzyme elevation was made.

**Conclusions:**

A feasible hypothesis to explain these rare effects could be that exaggerated adrenergic activity occurred on microcirculation, leading to an excessive peripheral vasoconstriction and subsequent ischemic damage. An individual predisposition is strongly suggested. A concurrence of events was probably responsible for the elevation of nonspecific tissue necrosis markers. Physicians and patients must be aware of these rare, but possible, adverse drug reactions.

## Introduction

Diosmin (3′,5,7-trihydroxy-4′-methoxyflavone-7-ramnoglucoside) is a natural bioflavonoid isolated from various plants or derived from the flavonoid hesperidin. Currently, it is used to relieve symptoms of venous insufficiency or capillary fragility, such as chronic venous insufficiency (CVI) and hemorrhoidal attacks. Self-medication with diosmin is frequent, since it is an over-the-counter (OTC) drug. Usual dosage of diosmin is 1000mg daily, in two divided doses. In the case of an acute hemorrhoidal attack, a loading dose of 3000mg per day for the first 4 days is needed, followed by 1000mg twice a day (bid) for 3 days [1].

In the case of CVI (which is characterized by structural/functional alteration of veins, and abnormal capillary hemodynamics), the main effects of diosmin are exerted on the walls of capacitance vessels and capillaries; it induces restoration of the normal venous tone, increasing resistance [2] and decreasing the permeability of microcirculation vessels [3]. Moreover, this drug also enhances drainage from lymphatic vessels [4-6]. Diosmin causes a significant decrease in plasmatic endothelial adhesion molecules and reduces neutrophil activation, thus providing protection against microcirculatory damage [7]. Pharmacokinetics and oral bioavailability of flavonoids change in relation to the qualitative composition of the extract or food: the two main sites of metabolism of flavonoids are the liver and the bowel. The O-methylation, the sulfation and the glucuronidation of the hydroxyl groups occurs in the liver, while flavonoids are subject to bacterial degradation in the colon. Following oral administration, diosmin is rapidly transformed into the active metabolite aglycone (diosmetin), by intestinal flora. Diosmetin is promptly absorbed by the gut mucosa, distributed, and finally extensively degraded to phenolic acids or their glycine-conjugated derivatives, which are eliminated with the urine. Approximately half of the dose is eliminated in the feces as unchanged diosmin or diosmetin [8]. In humans, diosmin pharmacokinetics based on oral administration to healthy volunteers showed a half-life of 31.5±8.6 hours, with maximum concentration (417±94.1ng/mL, for an oral dose of 10mg/kg) reached after 1 hour and followed by slow decrease after 2 hours [9]. The prolonged presence of diosmin in the blood suggests an enterohepatic circulation that is known to slow down the elimination of drugs. The same study also revealed that the distribution volume of diosmin is high (62.1±7.9L), and this may explain the low diosmin plasma levels, in comparison to the orally administered dose.

Short-term administration of diosmin is reasonably safe for most patients, even if it may cause mild dermatological, gastrointestinal, and autonomic side effects. The most common side effects are: pruritus, erythema, dermatitis, stomach and epigastric pain, nausea and diarrhea, dizziness. The first three commonest diosmin side effects as reported by the US Food and Drug Administration (FDA) [10] are: dermatological disorders, cardiac arrhythmias and hemolytic anemia.

The aim of this study is to describe and assess diosmin-related adverse events (AEs) reported by patients to their general practitioner. To the best of our knowledge, this is the first case report of diosmin-induced serum creatine phosphokinase (CPK) and lactic dehydrogenase (LDH) enzyme elevation during diosmin therapy. The aim of this study is to describe the cases and analyze the possible causes of these observed and unexpected adverse drug reactions (ADRs) not described in the Summary of Product Characteristics.

## Case presentation

Seventeen diosmin-related suspected ADR reports were collected during a period of about 4 years (from January 2008 to May 2012). Table 1 shows all patients’ features and concomitant medications. Our database contains data about 14 women and 3 men; all patients were Caucasian and aged between 23 and 81. Of these, 16 patients were taking the recommended therapeutic diosmin dose: diosmin alone 450mg bid (nine patients), and diosmin 450mg plus hesperidin 50mg three times a day (tid; eight patients), to treat acute hemorrhoidal attacks or CVI. Only one patient was taking a supratherapeutic dose of diosmin (900mg tid). Most of the reported side effects usually concern gastroenterological (stomach and epigastric pain, nausea and diarrhea) and dermatological (hives and itching) systems. All AEs were mild to moderate, no severe or life-threatening reactions requiring dose reduction or discontinuation of the treatment occurred.

**Table 1:** Patients’ characteristics

**Pt**	**Sex**	**Weight (Kg)**	**Age (years)**	**Drug**	**Posology**	**AEs**	**Dechallenge**	**Rechallenge**	**Concomitant therapy**
1	F	70	55	diosmin 450mg	1 bid	increased CPK, myalgia	yes*	yes	amiloride, hydrochlorothiazide
2	F	85	70	diosmin 450mg + hesperidin 50mg	1 tid	hives	yes		
3	F	65	80	diosmin 450mg + hesperidin 50mg	1 tid	hives	yes		
4	F	60	41	diosmin 450mg + hesperidin 50mg	1 tid	hives	yes		
5	F	65	81	diosmin 450mg + hesperidin 50mg	1 tid	diffused itching	yes		parnaparin, prednisone, citalopram, rabeprazole, acetylsalicylic acid, simvastatin, amlodipine
6	F	55	23	diosmin 450mg + hesperidin 50mg	1 tid	tachycardia	no*		local medication (for perianal fistulectomy)
7	F	75	36	diosmin 450mg	1 bid	epigastric pain, fainting	yes		lisinopril, acetylsalicylic acid
8	F	95	65	diosmin 450mg	1 bid	epigastric pain	yes	yes	amlodipine, telmisartan
9	F	68	71	diosmin 450mg	1 bid	epigastric pain	yes		
10	F	70	53	diosmin 450mg + hesperidin 50mg	1 tid	epigastric pain	yes		
11	M	70	43	diosmin 450mg + hesperidin 50mg	1 tid	diarrhea	yes		
12	M	65	43	diosmin 450mg	1 bid	diarrhea	no		
13	F	73	35	diosmin 450mg	1 bid	itching	yes		
14	F	80	69	diosmin 450mg	1 bid	epigastric pain	no		
15	F	77	66	diosmin 450mg	1 bid	asthenia, nausea	no		
16	M	80	79	diosmin 450mg	2 tid	increased LDH	yes		anal topical application, amlodipine
17	F	75	53	diosmin 450mg + hesperidin 50mg	1 tid	hives	yes		cefixime, rabeprazole, acetylsalicylic acid, carvedilol, amlodipine, levothyroxine, simvastatin

AEs, adverse events; bid, twice a day; CPK, creatine phosphokinase; F, female; LDH, lactic dehydrogenase; M, male; Pt, patient; tid, three times a day; *yes, means that the side effects resolved after the drug was stopped; *no, means that the event resolved before the drug was stopped.

Two cases aroused our attention because they are, to date, unique case presentations of acute, diosmin-related enzymes elevation (CPK and LDH).

Case 1 patient is a 55-year-old Caucasian woman with legs edema and swelling. She had been taking amiloride 5mg + hydrochlorothiazide 50mg, once daily, for several years to treat mild hypertension. A venous Doppler was performed, and right ostial saphenofemoral incompetence was diagnosed. Thus, the physician started diosmin 450mg bid treatment, since clinical features of this patient seemed not to be so alarming to require major interventions. Five days after starting diosmin treatment, the patient complained of worsening leg pain (diffuse myalgias), and, for this reason, at day 7, she quitted diosmin therapy, and her myalgias disappeared after 3 days. At day 29 she decided, without informing the physician, to take again diosmin at the same posology previously prescribed, but her myalgias reappeared (positive rechallenge) and after 2 days of treatment, she definitely gave up on diosmin therapy. Three days after, her physician prescribed her some blood tests. Her serum CPK dosage was the only out-of-range biochemical value (1500 IU/L; normal value 39 to 308 IU/L). Her physician excluded all other possible causes for myalgias and CPK elevation: she did not perform unusual heavy exercise; myocardial infarction was excluded by electrocardiogram, echocardiography, and cardiac enzymes measurement; besides she was taking neither statins, nor OTC drugs or herbal remedies. Thus, her physician diagnosed that diosmin was responsible for this AE. Two months after diosmin suspension, in a follow-up blood test, all evaluated hematology and serum chemistry parameters were within the physiological range, including serum CPK (value: 180 IU/L). The association between reported symptoms and signs, and diosmin therapy, according to the Naranjo scale [11], was given a score of 5 (probable).

Case 2 patient is a 79-year-old Caucasian man, who was diagnosed with acute hemorrhoidal attack. His symptoms were severe: rectal bleeding, mucous discharge, swelling around the anal area and significant pain, therefore the physician prescribed diosmin 450mg two tablets tid and the use of topical nifedipine (0.3g) plus lidocaine hydrochloride (1.5g). He has been taking amlodipine (10mg, daily) for 2 years, since he was suffering from hypertension. After 7 days of treatment with diosmin, hemorrhoidal symptoms gradually improved, and he fully recovered after 10 days. Despite healing, he kept on taking diosmin, at the same dosage, for another 21 days. After this period he quitted diosmin therapy, and received blood tests, as part of a routine check-up. Increased levels of LDH (1100 IU/L; normal value: 240 to 480 IU/L) were observed. It was the only out-of-range biochemical value. After 1 month, blood tests showed a normalization of LDH values (265 IU/L). In this case, rechallenge was not performed. The association between the accidentally observed LDH elevation and diosmin therapy, according to the Naranjo scale, was given a score of 3 (possible).

## Discussion

A comprehensive review of the literature using Medline/PubMed database was performed and a few cases of diosmin-related ADR reports were found. In most cases diosmin causes minor side effects, stomach and abdominal pain, diarrhea, dermatological disorders, and headache [12]. Few cases of hemolytic anemia have been reported by FDA; nevertheless, no published data are available [10]. In order to explain the mechanisms of the suspected diosmin-related ADRs, we verified if the AEs might be associated with diosmin pharmacokinetics. Despite a comprehensive and laborious review of literature, we were not able to hypothesize any pharmacokinetic correlation. It is not conceivable to suppose a longer enterohepatic recycling or alteration in the excretion of diosmin. Besides, we excluded, in both cases, any possible interactions between diosmin and concomitant antihypertensive therapies. In the Case 1 patient, the possibility of amiloride/hydrochlorothiazide-induced imbalance in electrolytes (a possible cause of myalgias and other disorders) was excluded because biochemical tests showed no abnormalities in the serum potassium concentration and all the other parameters were within the physiological range. Furthermore, positive rechallenge strengthens the hypothesis of a diosmin-related adverse reaction.

As regards to the Case 2 patient, the conditions that can raise LDH levels (heart failure, hypothyroidism, anemia, and lung or liver disease) were excluded. Since LDH is often used as a marker of tissue breakdown and can function as a marker for hemolysis, we took into account the possibility of hemolytic transient anemia, (already described as a possible diosmin side effect by FDA [10]) that could have occurred as a complication of the supratherapeutic dose of diosmin (900mg tid). Nevertheless the absence of anemia, elevation of indirect bilirubin and reticulocytosis allow us to exclude this hypothesis. Finally, errors in laboratory measurements were excluded.

Kumar *et al.*[13] reported a case of an intraventricular hemorrhage in a 77-year-old patient during a protracted diosmin therapy. The authors ascribed the event to the ability of diosmin to enhance the effects of noradrenaline on venous tone and in inhibiting platelets aggregation. The authors also hypothesized that prolonged use of diosmin had probably induced a chronic reinforcement of noradrenergic tone on choroid plexus microcirculation leading to increased intravascular pressure.

As reported by Sher *et al*. [14] and, more recently, reviewed by Middleton *et al.*[15], diosmetin, the active metabolite of diosmin, behaves as an antagonist of plasma membrane amine transporters at the molecular level, suggesting that inhibition of amine reuptake at peripheral sympathetic nerve terminals could lead to an increased vascular tone. However, as reported by Boudet and Peyrin [2], diosmin vasotonic effects could also be due to the inhibition of venous catechol-O-methyltransferase activity with a consequent decrease in the metabolism of noradrenaline at the vein wall; this mechanism of action could induce peripheral vasoconstriction mediated by reduced rate of noradrenaline metabolism. It could be hypothesized that enhanced adrenergic tone might induce peripheral vasoconstriction leading to ischemic muscles damage, associated with myalgia, and augmented levels of unspecific tissue necrosis enzymes (CPK and LDH).

In the Case 1 patient, chronic leg edema, probably due to CVI, could have caused higher baseline intravascular and intracapillary pressures, making tissues more vulnerable to trauma from vasoconstriction. In the Case 2 patient, an acute hemorrhoidal attack could have been severe enough to cause tissue ischemia and subsequent LDH elevation without hemolysis; prolonged diosmin therapy could have enhanced tissue necrosis due to its vasotonic effects, acting as a concurrent cause of LDH increase. It seems that the association between diosmin and the reported AEs should be ascribed to an individual and subjective predisposition to the adrenergic effects of diosmin, and/or to the presence of comorbidities.

## Conclusions

To the best of our knowledge, these are first cases of increased serum CPK and LDH levels during diosmin therapy to date reported.

We ascribe these reported ADRs to a concurrence of events: an individual predisposition to the vasotonic effects of diosmin, and the presence of severe comorbidities that may have enhanced noradrenaline tone in some predisposed patients.

Drugs containing diosmin are frequently prescribed by general practitioners. Diosmin is usually considered safe because of its natural origin, and its phytochemical and pharmacological features. Physicians and patients must be aware of these rare, but possible ADRs.

Further studies are needed to investigate in more detail the underlying causes of these ADRs.

## Consent

Written informed consent was obtained from both patients for publication of these two case reports. A copy of the written consent is available for review by the Editor-in-Chief of this journal.

## Abbreviations

ADR: Adverse drug reaction; AE: Adverse event; bid: Twice a day; CPK: Creatine phosphokinase; CVI: Chronic venous insufficiency; FDA: Food and Drug Administration; LDH: Lactic dehydrogenase; OTC: Over-the-counter; tid: Three times a day.

## Competing interests

The authors declare that they have no competing interests.

## Authors’ contributions

GM performed the analysis of data, and drafted the manuscript. SL provided language help and writing assistance. MLZ carried out a literature review about the topic. CF participated in data collecting. AS collected and reported adverse drug reactions data. AM conceived of the study, and participated in its design. FM participated in its design and coordination and helped to draft the manuscript. All authors read and approved the final manuscript.
